# Additive Manufacturing with Thermoplastic Collagen

**DOI:** 10.3390/polym14050974

**Published:** 2022-02-28

**Authors:** Enno Klüver, Marit Baltzer, Axel Langer, Michael Meyer

**Affiliations:** FILK Freiberg Institute gGmbH, D-09599 Freiberg, Germany; marit.baltzer@filkfreiberg.de (M.B.); axel.langer@filkfreiberg.de (A.L.); michael.meyer@filkfreiberg.de (M.M.)

**Keywords:** collagen, thermoplast, additive manufacturing

## Abstract

Thermoplastic collagen is a partially denatured collagen powder which can be processed by thermoplastic methods such as extrusion and injection molding, but was hitherto not adapted for the use in additive manufacturing (AM) techniques. This paper describes the first successful application of collagen/water/glycerol mixtures in an AM process using a BioScaffolder 3.2 from GeSiM mbH. Strands of molten collagen were deposited onto a building platform forming differently shaped objects. The collagen melt was characterized rheologically and optimal processing conditions were established. The technique includes the use of supporting structures of PLA/wood composite for samples with complex geometry as well as post-processing steps such as the removal of the supporting structure and manual surface smoothing. The manufactured objects are characterized concerning water solubility, swelling behavior and compressibility. Possible applications are in the non-medical sector and include collagen-based pet food or customized organ models for medical training.

## 1. Introduction

Among all preparations of collagen, native or denatured, thermoplastic collagen takes a unique position. It comprises a dry powder, which can be processed by common thermoplastic techniques, such as extrusion or injection molding. Thermoplastic collagen is produced from collagenous tissues, such as bovine or porcine hide, after dehairing. The production process includes a denaturation step, drying and milling [1]. A suitable collagen mixture for thermoplastic processing contains water as a mandatory plasticizer and other optional additives, such as glycerol, dyes or inorganic salts. Extrusion or injection molding is operated at moderate temperatures (90–100 °C) and results in versatile products, such as pellets, threads, sheets, films or molded parts [1,2].

Until now, thermoplastic processing of collagen was limited to extrusion and injection molding. The simultaneous impact of thermal energy and mechanical stress, which is inherent to these techniques, is considered a prerequisite for the melting of collagen-based compositions. Nowadays, synthetic thermoplastic polymers are widely used in additive manufacturing processes (AM), enabling fast and customized production of various products. The adaptation of thermoplastic collagen to AM techniques is therefore a great concern. There is no evidence in the literature of the use of thermoplastic collagen in AM processes. The use of this powder as a bio-based material in thermoplastic additive manufacturing will open new applications, products and markets.

A large amount of different AM methods have been established in recent decades [3,4,5,6,7,8,9,10,11,12]. From this variety, fused deposition modeling techniques (FDM) are most suitable for thermoplastic collagen preparations, although they were not directly transferable. The simplest variant, the fused filament fabrication (FFF), is established today in industries and households. It is commonly called “3D printing” and uses a filament of thermoplastic polymer, which is molten by heat and deposited in the form of a molten strand onto a building platform. The desired object is built up layer by layer via movement of the printing unit in x-, y- and z-directions [13,14,15]. This setup is not applicable for thermoplastic collagen, since it is not possible to melt a collagen filament only by heating, but a mechanical stress component is always needed. The Arburg Freeformer^®^ is an extrusion-based commercial variant, which generally could work for thermoplastic collagen [16]. However, its functional principle involves the deposition of small molten droplets instead of strands, which is beyond the rheological scope of thermoplastic collagen. An additive manufacturing process suitable for thermoplastic collagen uses a printing system with a pressure-driven, heatable cartridge, which is primarily designed for bioprinting techniques. Bioprinting methods are similar to FDM methods in a way that they deposit a given material, which may be an aqueous solution, hydrogel or dispersion of biopolymers or even cells, and which is provided in a cartridge, in the form of strands or droplets onto a building platform. Usual applications include the formation of three-dimensional scaffolds for medical applications or tissue engineering, often demanding additional stabilization by cross-linking procedures after printing [17,18,19,20,21,22,23,24,25,26]. The term bioprinting is often associated with medical applications, which can be realized also with medical grade thermoplasts such as polycaprolactone, polylactic acid or polypropylene, often in combination with other polymeric components such as hyaluronic acid, natural fibers or active ingredients [27]. The technical setup of the bioprinter BioScaffolder 3.2 (GeSiM mbH, Radeberg, Germany) with a cartridge, nozzles with varying diameter, and a pressure-based and moveable metering unit is appropriate for the processing of thermoplastic collagen preparations. The temperature applied to the cartridge and the mechanical stress from the pressure-driven piston are sufficient to melt the collagen composition and to produce a strand of collagen melt. Native collagen is already used in bioprinting techniques in the form of a solution or hydrogel, often combined with viable cells, for applications in regenerative medicine, tissue engineering or lab-on-a-chip systems [26,27,28,29,30,31,32,33,34,35,36,37,38]. The drawback of native collagen hydrogels in bioprinting is their low mechanical stability, slow gelation rate and often inhomogeneous cell distribution [23]. Thermoplastic collagen is mechanically more stable and the solidification of the melt is faster, resulting in a higher dimensional stability. Thus, thermoplastic collagen, although not comprising completely native collagen, offers great potential for medical applications, such as implants, regenerative medicine or tissue engineering, where a biocompatible material with medium mechanical stability is required. This paper describes the successful adaptation of thermoplastic collagen as a novel bio-based material in thermoplastic additive manufacturing applications. It focusses on the technical conditions and the basic material parameters of the starting material as well as of the printed objects.

## 2. Materials and Methods

### 2.1. Materials

Thermoplastic collagen was produced in-house from bovine skin. Raw skins were dehaired in a standard liming process with sodium sulfide at pH 12. The dehaired pelts were dried at 80 °C in a rack dryer in order to denature and dry the material simultaneously. The dry pelts were ground to a fine powder (cutting mill SM2000, Retsch, Haan, Germany, and turborotor mill, Görgens Engineering GmbH, Dormagen, Germany) and sieved. The fraction with grain sizes below 0.25 mm was used in the AM process.

Glycerol (technical grade, 99%) was purchased from AppliChem (Darmstadt, Germany), the PLA/wood composite (10% wood content) was from Flashforge 3D Printer (Zhejiang, China). Dyes E122 (Azorubin) and E155 (Brown HT) were from Ruth GmbH (Bochum, Germany).

### 2.2. Technical Processes

Additive manufacturing process. STL data files for the objects to be printed (cylinder, cube, egg-shaped) were designed using the software Materialise Magics 23.01 (Materialise GmbH, Gilching/München, Germany). Supporting structures were realized using the slicer software Ultimate Cura 4.8.0 (Ultimaker B. V., Utrecht, The Netherlands). Additive manufacturing was performed on a BioScaffolder 3.2 (GeSiM mbH, Radeberg, Germany). The equipment contains a 20 mL cartridge, heatable up to 200 °C. Material is pressed through a nozzle (diameter 0.25 or 0.40 mm) using a piston driven by air pressure (up to 5.6 bar). The building platform is 20 × 20 cm in size. Printing velocity and layer distance in z-direction were established manually for each mixture. The optimized set of parameters were: cartridge temperature: 90 °C, air pressure: 5 bar, printing velocity: 3 mm/s, nozzle diameter: 0.25 mm, and layer distance in z-direction: 0.4–0.45 mm. Objects with more complex geometry were manufactured with the help of a supporting structure from PLA/wood composite. The support was manufactured with a parallelly mounted cartridge under conditions suitable for the polymer (temperature 190 °C, pressure 0.5 bar, printing velocity 40 mm/s).

Post-processing treatment. Supporting structures from PLA/wood were removed manually. The rough surface of as-printed objects was smoothed manually with warm water.

### 2.3. Material Characterization

Moisture content. A sample of collagen powder or printed object was weighed before and after drying (102 °C, 5 h). The moisture content was calculated from the mass difference in relation to the initial sample weight.

Protein content. The total nitrogen content of collagen powder was determined by titration of ammonia after acidic hydrolysis, according to the standard method DIN 53308.

Water solubility. A sample of collagen powder or printed object (ca. 1 g) was agitated in an Erlenmeyer flask with deionized water (50 mL) for 16 h at 4 °C or for 6 h at 60 °C, respectively. Insoluble parts were filtrated and the dry mass of the filtration residue was determined. The water solubility was calculated as the ratio of the dry masses of filtration residue and weighed sample. All measurements were performed in duplicates.

Swelling in water. A sample of collagen powder or printed object (ca. 1 g) was agitated in an Erlenmeyer flask with deionized water (50 mL) for 16 h at 4 °C or for 2 h at 60 °C, respectively. The sample was filtrated and weighed. The water swelling was calculated as the ratio of the difference between wet weight and dry mass in relation to the dry mass. All measurements were performed in duplicates.

DSC measurements. A sample of collagen powder was soaked in phosphate buffer (pH 7) for 2–5 h. DSC measurement was performed in a differential scanning calorimeter DSC 1 STAR (Mettler Toledo, Germany) using an aluminum crucible in a temperature interval from 0–120 °C with a heating rate of 5 °C/min. The onset and maximum of peaks were determined from the graphical display of heat flow vs. temperature.

Viscosity of TC melts. Collagen-based mixtures were prepared with varying water and glycerol content. Mixtures were defined on weight basis in relation to collagen: TC: water:glycerol = 1: 0.9: 0.25, 1: 1.0: 0.25, 1: 1.1: 0.25, 1: 1.2: 0.25, 1: 1.3: 0.25, 1: 1.2: 0.35, 1: 1.2: 0.45, 1: 1.2: 0.55, and 1: 1.2: 0.65. They were processed using a single screw extruder Rheomex 302 (L/D = 33, four heating zones) and extruded through a slit die (w = 20 mm, h = 0.8 mm) provided with two pressure sensors in a distance Δl = 5 cm (PolyLab OS system, ThermoFisher Scientific, Karlsruhe, Germany). The extrusion temperature was 100 °C at extruder exit and die. The shear rate γ was regulated via the rotating speed of the extruder screw and calculated from the measured mass flow ṁ using the formula γ = 6·ṁ /(ρ·w·h^2^) with a density ρ of 1.2 g/cm^3^. The shear rate covered a range from 17 to 593 s^−1^, depending on the individual mixture. The melt viscosities were calculated from the measured pressure differences Δ*p* using the formula η = (w·h·Δ*p*)/(2·(w + h)·Δl). Multiple measurements were averaged and displayed graphically using double logarithmic diagrams. The melt flow index MFI of the collagen melt in the 3D printing process was calculated under optimum printing conditions using the printing velocity v and strand diameter d by the formula MFI = 600·π·(d/2)^2^ ρ [g/10 min].

Compressive strength. The compressibilities of the printed objects and extruded strands were determined using a hydraulic press Z010 (Zwick Roell GmbH, Ulm, Germany). Cylindrical objects (diameter 10 mm, height 10 mm) were compressed until they reached 50% of their initial height, and the pressure was detected.

## 3. Results and Discussion

Thermoplastic collagen powder consists of more than 87% of protein with low mineral content (see Table 1). Non-detected components may include carbohydrates. DSC measurements show two small peaks with onset temperatures at 18.4 and 36.4 °C, respectively, indicating a material with native and denatured fractions (the denaturation temperature of native collagen being ca. 37.5 °C [39]). As a partially denatured collagen preparation, the powder shows a significant water solubility even in cold water (4 °C) and a considerable swelling behavior, which is important for additive manufacturing applications.

Preparations of thermoplastic collagen for application in thermoplastic processes usually include water as a mandatory plasticizer [1]. In fact, it is not thermoplastic collagen itself which can be considered thermoplastic, but the system collagen/water, wherein water comprises 40–60% of the whole mass. Extrudates of only collagen and water desiccate while stored at airbecome stiff and brittle. Therefore, glycerol is usually added as a permanent plasticizer, providing long-term flexibility [1].

Direct mixing of the components collagen, water and glycerol resulted in a gum-like dough, which was not entirely homogeneous and could not be loaded into the printer’s cartridge without the inclusion of air, which subsequently led to an inhomogeneous discharge of the melt from the die. Therefore, the mixture was extruded prior to the printing process. This pre-extrusion served as a homogenizing step for the mixture and was performed using a circular die with the same diameter as the printer’s cartridge. Sections of the extruded strand were thus used as apportioning sticks for the cartridge, and the material could be loaded without air inclusion.

**Figure 1:**
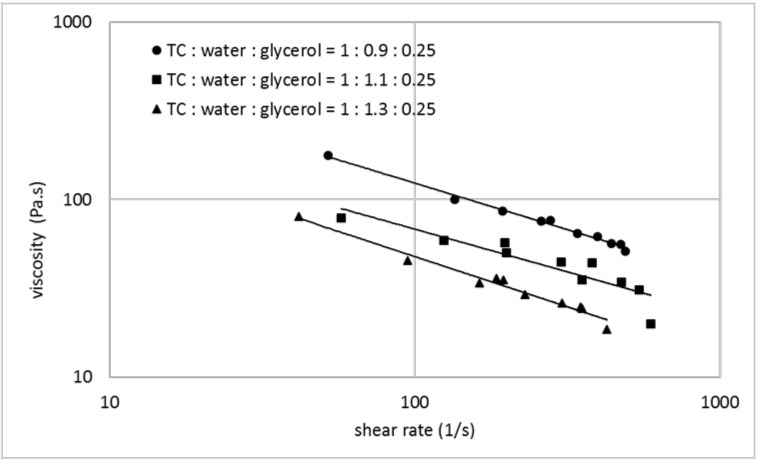
Melt viscosity vs. shear rate for collagen/water/glycerol mixtures with varying water content (mass fraction of water in relation to collagen: 0.9 (circles), 1.1 (squares) and 1.3 (triangles)) in double logarithmic representation.

**Figure 2:**
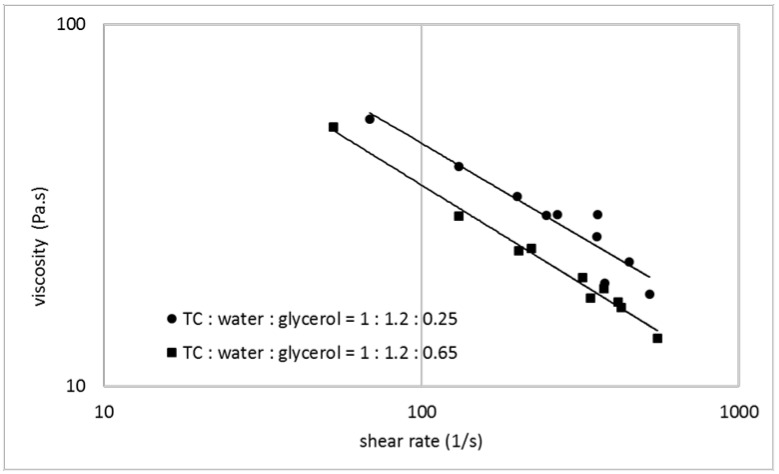
Melt viscosity vs. shear rate for collagen/water/glycerol mixtures with varying glycerol content (mass fraction of glycerol in relation to collagen: 0.25 (circles) and 0.65 (squares)) in double logarithmic representation.

**Figure 3:**
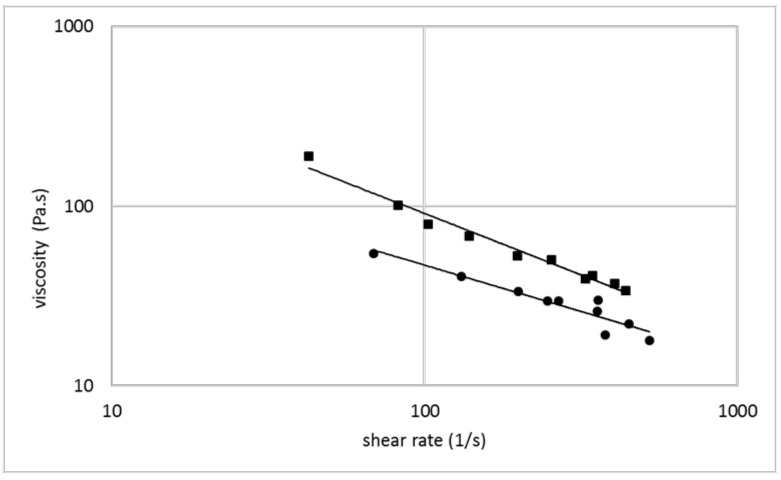
Melt viscosity vs. shear rate for collagen/water/glycerol mixtures (1:1.2:0.25) for extrusion of a pristine mixture (circles) and a pre-extruded mixture (squares) in double logarithmic representation.

The ability of a thermoplastic material for use in an additive manufacturing process depends on its melt viscosity and the possibility to influence and control this parameter. Figure 1, Figure 2 and Figure 3 show the melt viscosity of typical collagen/water/glycerol mixtures in dependence on the shear rate in an extrusion process.

The curves represent shear thinning behavior, typical for thermoplastic materials. The melt viscosity decreases with increasing water content, whereupon even small changes in the water content show a significant effect (Figure 1). Glycerol shows a similar effect, but compared with water, to a lesser extent (Figure 2). When a collagen/water/glycerol mixture is pre-extruded and then extruded a second time, the melt viscosity in the second extrusion increases by an average of 60% (Figure 3). This effect can be explained by a reduced water content due to the extrusion process. Analytical data showed a water content of 43.3% before extrusion vs. 40.9% after extrusion, corresponding to a loss of ca. 6% of the contained water. This fact is consistent with literature data, where extrusion is reported to be responsible for a loss of water between 7 and 15% [1]. These findings illustrate the main influence of the water content on the melt viscosity of collagen-based mixtures. It has an important impact on the collagen melt and can be conveniently controlled. Common parameters in additive manufacturing with thermoplastic polymers are the molecular weight and weight distribution. In the case of thermoplastic collagen, this parameter is not applicable. The molecular weight of thermoplastic collagen is not accessible by common analytical methods, since the powder cannot be dissolved completely. The source for thermoplastic collagen is a grown tissue, which represents a cross-linked elastomeric network with a molecular weight of several million. Collagen powder is derived from this high-molecular protein network by unspecific degradation, resulting in fragments with unknown mass distribution. Even if the molecular weight was known, this parameter is hardly controllable in the production process. There are distinct differences to gelatin (only partial solubility, no gelling behavior, higher denaturation temperature) which indicates that thermoplastic collagen is less degraded than gelatin, but the particular molecular weight remains unknown.

Another important parameter is the stickiness of the molten strand which is a prerequisite for the adhesion between the strands of successive layers and hence for the compactness and stability of the manufactured object. Water and glycerol content influence the stickiness, which was evaluated qualitatively. Several mixtures with varying water and glycerol content were investigated and rated concerning their processability in a bioprinter (melt viscosity, stickiness of extruded strand, expansion rate). The optimal composition appeared to be collagen/water/glycerol = 1/1.2/0.55. A molten strand of this mixture was ideal for deposition on the building platform, and the surface of the strand was slightly sticky which enabled a seamless fusion of a deposited layer with the subjacent one. A printing velocity of 3 mm/s was optimal for this material. The calculated melt flow index MFI under these conditions was 0.27 g/10 min, which is consistent with literature data of protein melts [40].

**Figure 4:**
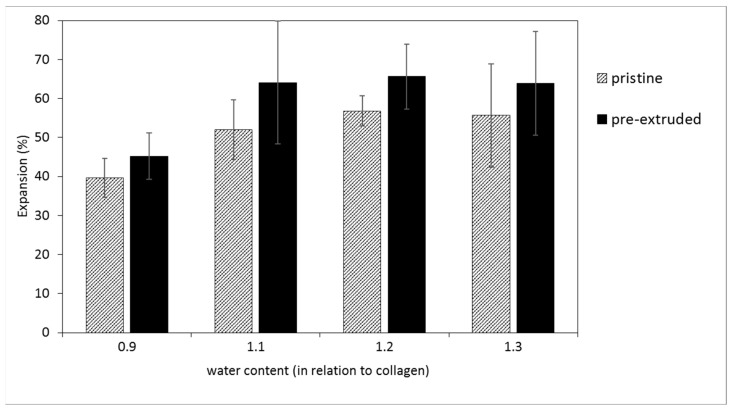
Expansion of collagen strands after the printer’s nozzle in relation to the nozzle diameter (0.25 mm) for pristine and pre-extruded mixtures in dependence on the water content (0.9, 1.1, 1.2, and 1.3 in relation to collagen, glycerol content 0.25 throughout).

It was observed that extruded strands of collagen/water/glycerol mixtures expand directly after the nozzle resulting in a larger diameter than the nozzle diameter. Depending on the water content, the expansion rate for mixtures with a 0.25 content of glycerol lies between 39 and 57% (in relation to the nozzle diameter) (see Figure 4). When the pre-extruded apportioning sticks, i.e., an already compacted material, are used for printing, the expansion is even higher (51–66%). This effect must be taken into account when adjusting the layer distance in the printing process. The expansion for the optimal composition was 72%, and consequently the layer distance in z-direction was adjusted to 0.4–0.45 mm.

For the additive manufacturing of objects with more complex geometry, supporting structures are needed in order to realize undercuts, gaps or hollows. With most synthetic polymers, water-soluble polymers are usually used for supporting structures, which can be dissolved easily after the printing process by inserting the object into the water for a given time. In the case of thermoplastic collagen, the approach was opposite. Due to the partial water solubility of collagen itself and the high water content of the collagen/water/glycerol mixtures, a supporting structure must be formed from a material, which is soluble in a non-aqueous solvent. A second criterion is the melting temperature, which must be compatible with the cartridge of the bioprinter. The used bioprinter in this study allowed heating up to 200 °C. PLA/wood was chosen from several variants since it meets both criteria. It is soluble in dichloromethane, which does not affect collagen, and the melting temperature lies between 180 and 220 °C, which can be afforded by the bioprinter. However, for potential medical applications a dichloromethane soluble material may be not the optimal choice; therefore, further alternative materials will be evaluated in future research.

**Figure 5:**
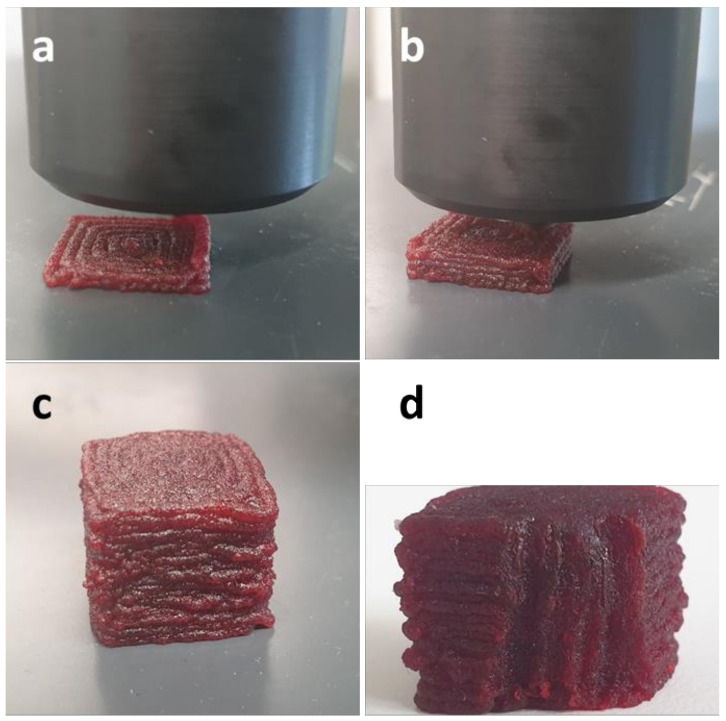
Additive manufacturing of a cubic object with a bioprinter from a collagen/water/glycerol mixture (colored with dyes E122 and E155 for optical reasons); (**a**–**c**) advancing printing process, (**d**) truncated cube showing the inner structure.

**Figure 6:**
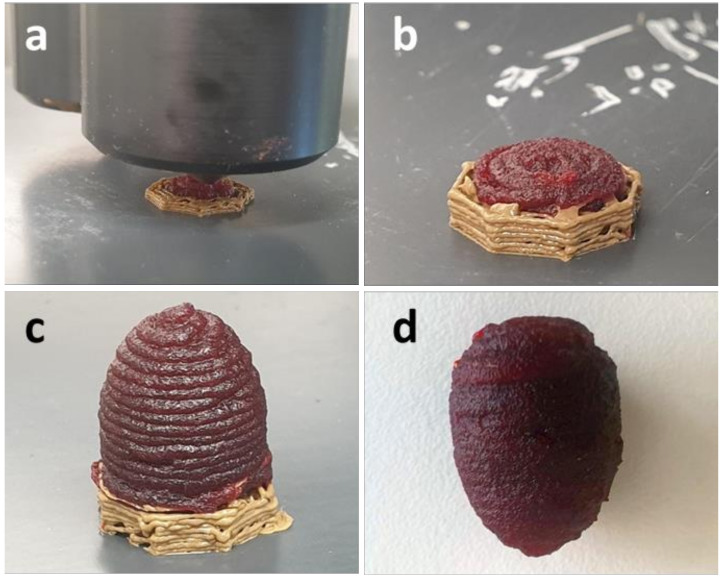
Additive manufacturing of an egg-shaped object with a bioprinter from a collagen/water/glycerol mixture (colored with dyes E122 and E155 for optical reasons) and with a supporting structure from PLA/wood; (**a**–**c**) advancing printing process, (**d**) object after the removal of the supporting structure and manual smoothing of the surface.

The process of additive manufacturing of samples from a collagen/water/glycerol composition is demonstrated in Figure 5 and Figure 6 for a simple cubic object and an egg-shaped object, which demands a supporting structure. Dyes (E122 and E155) were added to the mixture (0.08% each) for a better optical representation and differentiation from the yellowish supporting structure. The surfaces of the as-printed samples are relatively coarse, due to the expansion of the molten strands resulting in a reduced resolution. The surface could be smoothed by manual treatment with warm water. The effect is shown in Figure 6d.

**Figure 7:**
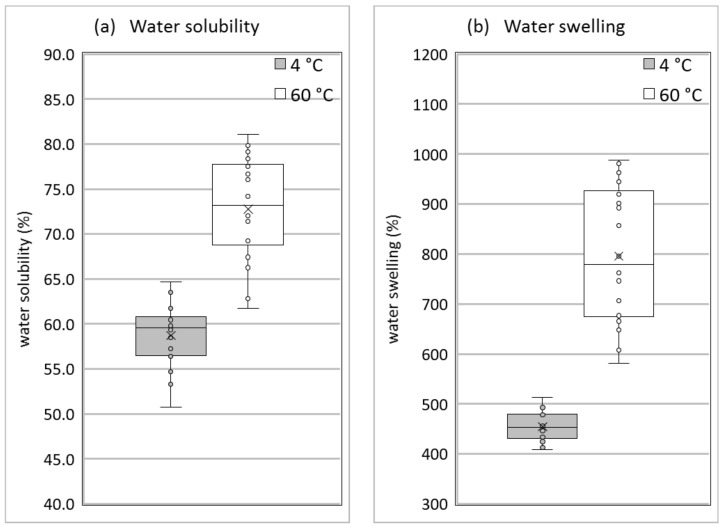
Box plots of water solubility (**a**) and water swelling (**b**) for all investigated collagen/water/glycerol mixtures at 4 °C and 60 °C.

For potential applications of collagen-based products, their stability against moisture and mechanical impact is an important factor. The stability of collagenous materials against water is relatively low. Figure 7 presents a summary of the water solubility and the degree of swelling in water at 4 °C and 60 °C for all measured compositions. They depend in detail on the water and glycerol content, but it is clearly visible that in any case collagen-based samples are significantly soluble in water (above 50% even at a low temperature) and show a considerable degree of swelling (400% and higher). This is due to the partial solubility of collagen itself (see Table 1) and the high water and glycerol content of the compositions. This moisture sensitivity is material inherent and must be taken into account when evaluating potential application fields.

**Figure 8:**
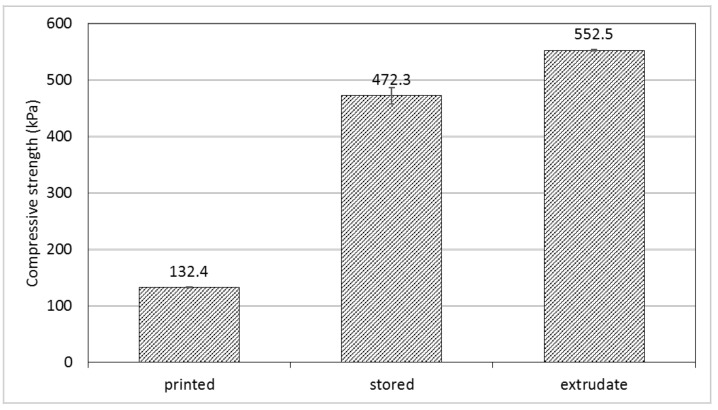
Compressive strength (kPa) of printed objects (collagen/water/glycerol = 1: 1.2: 0.55, pre-extruded): printed—directly after 3D printing, stored—after 24 h in a closed bag, extrudate—after extrusion through an extruder.

It is often discussed as a disadvantage of fused filament fabrication that manufactured objects may be less dense and therefore mechanically less stable than their counterparts produced by extrusion or injection molding [9,41]. Cylindrical objects from collagen/water/glycerol mixtures were characterized concerning their compressibility and compared with a cylindrical strand, produced with a conventional extruder, which was expected to be more compact. Freshly printed objects are still warm and gum-like, and their compressive strength is relatively low (see Figure 8). When stored at ambient conditions, they desiccate to a certain extent, the glycerol content preventing complete desiccation. Thus, when the compressibility of a sample was measured after 24 h of storage, the compressive strength has increased by 3.5 times. This is only 16% lower than the compressibility of an extrudate of the same composition. Thus, collagen-based objects produced by additive manufacturing show a compactness comparable with extruded samples. This is also illustrated by the insight into the inner structure of a printed cube in Figure 5d, which appears homogeneous and non-porous.

A general issue with collagenous materials is the fact that the feedstock (animal hide) is a natural product, which implies a certain fluctuation of material parameters. As a consequence, equal collagen-based mixtures from different batches may behave slightly differently. In fact, the expansion rate of the mixtures with the same composition depended on the individual collagen powder and, consecutively, the layer distance in z-direction had to be adjusted manually, which is a drawback for a fully automated process. The production of larger batches of collagen powder with averaged and more homogeneous properties is therefore a concern for future work.

## 4. Conclusions

Thermoplastic collagen powder was successfully adapted for additive manufacturing using a bioprinter with a heatable cartridge. Compositions of collagen, water, and glycerol were deposited in the form of molten strands resulting in compact objects. Samples with more complex geometry, such as egg-shaped objects, were constructed using a supporting structure from PLA/wood composite. Due to the expansion of the molten strands after the printer’s nozzle, the resolution of the printed objects is reduced, and the coarse surface needs manual smoothing treatment with warm water. The samples are compact with low to medium stability concerning moisture and mechanical stress, as expected for collagen-based products. Important issues, which will be investigated and optimized in future research, include the production of more homogeneous master batches, improvement of the resolution, the effects of other additives, such as mineral components or cross-linking agents, the evaluation of alternative materials for supporting structures, and the manufacturing of objects with more complex geometry or hollow structures.

## Figures and Tables

**Table 1:** Material characterization of thermoplastic collagen powder.

Parameter	Unit	Value	Standard Deviation
Dry matter (d.m.)	%	93.04	3.07
Mineral content	% d.m.	0.42	0.04
Protein content	% d.m.	87.07	0.37
Denaturation temperature			
Peak 1 (onset)	°C	18.44	0.23
Peak 2 (onset)	°C	36.36	1.51
Water solubility at 4 °C	% d.m.	8.42	1.24
Water solubility at 60 °C	% d.m.	39.69	1.83
Water swelling at 4 °C	% d.m.	9.21	1.44
Water swelling at 60 °C	% d.m.	65.90	5.12
